# Central Diabetes Insipidus: An Acute Manifestation of COVID-19 Infection

**DOI:** 10.7759/cureus.43884

**Published:** 2023-08-21

**Authors:** Sharanya Suresh Kumar, Kiran Kumar, Sneha Venkataramani, Naail Mohammed Ghazi

**Affiliations:** 1 General Medicine, Thumbay University Hospital, Ajman, ARE; 2 Internal Medicine, Thumbay University Hospital, Ajman, ARE; 3 College of Medicine, Gulf Medical University, Ajman, ARE

**Keywords:** urine osmolality, central diabetes insipidus, desmopressin, plasma osmolality, covid-19

## Abstract

In recent years, there has been a rise in the number of COVID-19 cases and its complications. Central diabetes insipidus (central DI) is a rare but treatable manifestation of acute COVID-19 infection. This case reports the rapid onset of central DI in a 35-year-old male in less than two weeks post-COVID-19 infection. He made a complete recovery post-administration of desmopressin within one month. Prompt diagnosis, treatment, and periodic follow-up are hence the cornerstones of a successful recovery for a patient with central DI post-COVID-19 infection.

## Introduction

COVID-19 or SARS-CoV-2 is an enveloped, non-segmented positive-sense RNA virus that belongs to the beta-coronaviridae family. It predominantly causes respiratory infection with the potential to cause severe pneumonia and ARDS with significant morbidity and mortality [1]. However, COVID-19 can cause extrapulmonary manifestations including cardiovascular, renal, thrombotic, gastrointestinal, neurological, and endocrinological symptoms. Data on both the endocrine imbalance during the phase of the infection and upon complete recovery remains largely unexplored and is still under study.

We present a case report of a young gentleman who had come with complaints of lower respiratory tract infection, but subsequently developed central diabetes insipidus (central DI) and made a complete recovery within one month, only 12 days after contracting the SARS-CoV-2 virus.

This case report was previously presented as a poster at the 13th Emirates Diabetes & Endocrine Congress (EDEC 2023).

## Case presentation

**Figure 1:**
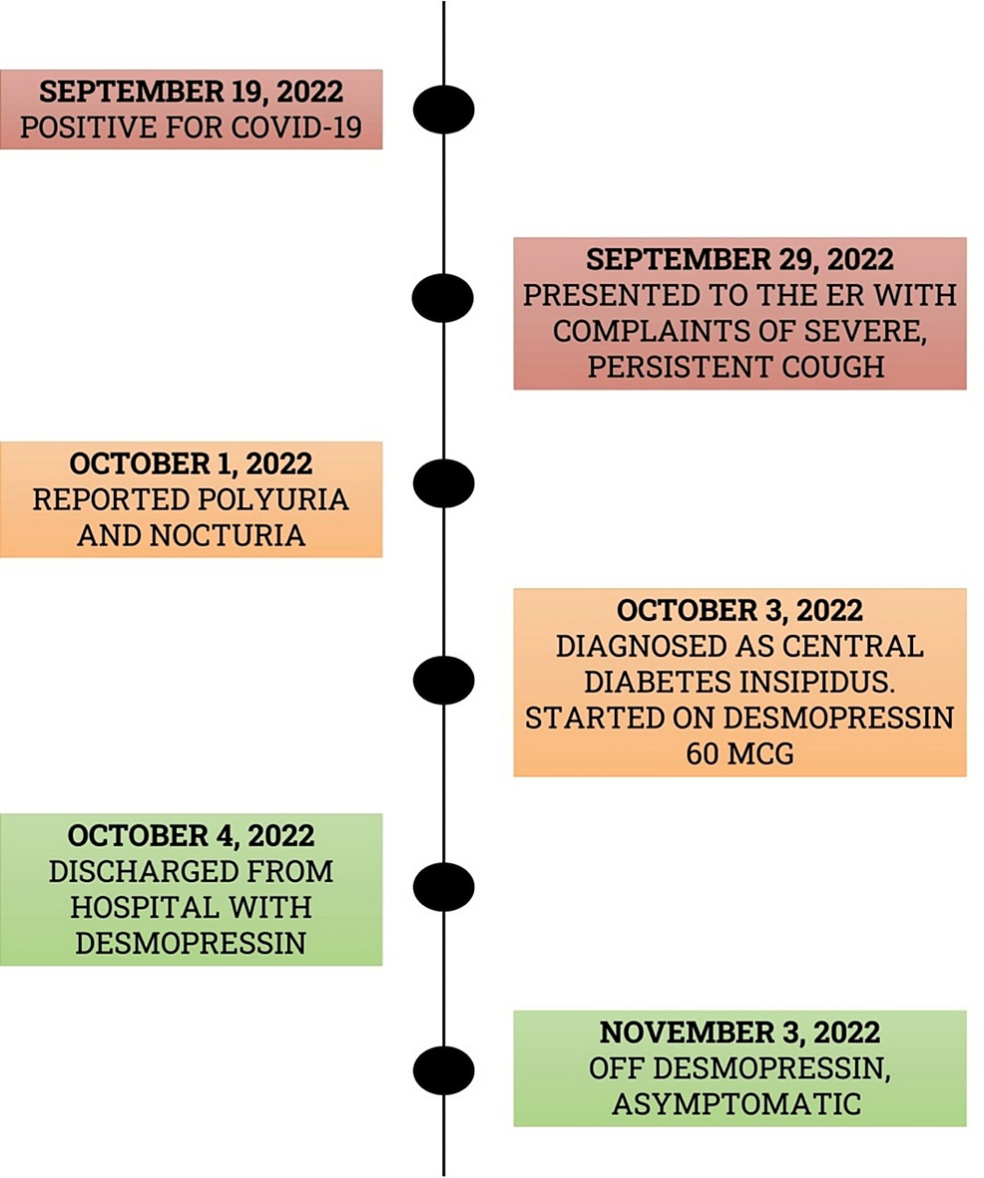
Timeline of our patient's presentation

Our patient, a 35-year-old male with no comorbidities presented to us on 29th September 2022 with complaints of severe and persistent cough of four days duration. He tested positive for COVID-19 by PCR on 19th September 2022. The patient had mild upper respiratory symptoms for the initial three days of COVID-19 diagnosis and recovered with home isolation (Figure 1). During the last four days before admission, his cough had increased and was persistent throughout the day. It was aggravated on exertion and was associated with minimal sputum production. There was neither fever nor headache associated. He denied any wheezes or dyspnea. The patient’s bowel and bladder functions were normal. In view of his continuous cough, he was advised admission for further evaluation and management. On general examination, low-grade fever was noted. Respiratory examinations were significant for harsh breath sounds; other systemic examinations revealed normal findings. The patient's initial laboratory evaluation (complete blood count, c-reactive protein, and kidney and liver function tests) was normal. The patient was treated with IV antibiotics, nebulization, cough suppressants, and proton pump inhibitors.

On the morning of 1st October 202,2 i.e., on his third day of hospital stay, the patient complained of frequent urination along with passing large volumes of clear urine and eight to 10 episodes of nocturia the previous night. His fasting blood sugar was 91 mg/dl and HbA1c was 5.1%, thus ruling out diabetes mellitus. History ruled out primary polydipsia and head trauma. A suspicion of diabetes insipidus was considered and the patient underwent 24-hour urine collection to check for urine osmolality along with urine sodium, urine chloride, urine potassium, and urine glucose. Serum osmolality, serum electrolytes, serum creatinine, and serum urea nitrogen were also measured. He was also evaluated for pituitary hormones like ACTH, cortisol, and thyroid function panel.

Results showed that a 24-hour urine volume of 4.2 liters, 24-hour urine osmolality of 256.6 mOsm/kg (normal reference: 500 to 800 mOsm/kg), 24-hour urinary sodium of 252 mmol, and 24-hour urinary chloride was 281.4 mmol. Serum osmolality was 285.7 mOsm/kg (normal reference: 275-295 mOsm/kg) and spot urine sodium was 90 mmol/L (normal 20-230 mmol/L). His 24-hour urine potassium, creatinine, urea nitrogen, and glucose along with his pituitary hormones (ACTH, cortisol, and thyroid stimulating hormone (TSH)) were in the normal range (Table 1). The patient was diagnosed with central DI, and he was started on desmopressin 60 mcg sublingual tablet at night on 3rd October. A dramatic response with a one-day desmopressin dose was noted, along with a reduction in the frequency of nocturia and day-time polyuria. The patient was discharged on 4th October and advised to continue 60 mcg of sublingual desmopressin. He continued the treatment for three weeks, and his dose was subsequently tapered to half a tablet for a week and then stopped. The patient followed up with the physician and reported a complete resolution of symptoms. His urine output was controlled and appropriate and hence his medications were discontinued, and he was asked to follow up with the physician periodically.

**Table 1:** Laboratory investigations of our patient CRP, c-reactive protein; HbA1c, glycated hemoglobin; TSH, thyroid stimulating hormone; ACTH, adrenocorticotrophic hormone

Tests Conducted	Patient’s Values	Reference Range
Hemoglobin	15.7 g/dL	13-17 g/dL
White blood count	8.7×10^3^/uL	4.0-10×10^3^/uL
Platelets	399×10^3^/uL	150-410×10^3^/uL
CRP	5.1 mg/L	<5 mg/L
Fasting blood sugar	91 mg/dL	<100 mg/dL
HbA1c	5.1%	<5.7%
TSH	2.788 uIU/mL	0.450-5.330 uIU/mL
Free T4 (thyroxin)	9.60 pmol/L	7.86-14.41 pmol/L
Serum sodium	139.4 mmol/L	136.0-145.0 mmol/L
Serum potassium	4.2 mmol/L	3.5-5.1 mmol/L
Serum urea	29.38 mg/dL	17.00-43.00 mg/dL
Serum creatinine	1.07 mg/dL	0.67-1.17 mg/dL
Serum ACTH	7 pg/mL	≤46.0 pg/mL
Serum cortisol (8 AM)	37.08 nmol/L	Morning hours (7-10 AM): 185-624 nmol/L; afternoon hours (4-8 PM): <276 nmol/L
Total urine volume in 24 h	4.2 L	
Serum osmolality	285.7 mOsm/kg	275-295 mOsm/kg
24-hour urine osmolality	256.6 mOsm/kg	500-800 mOsm/kg
24-hour urine sodium	252 mmol/24 h	40-220 mmol/24 h
24-hour urine chloride	281.4 mmol/24 h	110.0-250.0 mmol/24 h
24-hour urine glucose	462 mg/24 h	<500 mg/24 h

## Discussion

Published literature suggests that COVID-19 can potentially affect the endocrine and nervous systems; one such manifestation of neuroendocrine pathology secondary to COVID-19 is central DI [2-4].

Various pathophysiologic mechanisms by which central DI secondary to COVID-19 has been described. Chigr et al. confirmed the presence of ACE2 expression in the paraventricular nucleus, thus making it a target for SARS-CoV-2 [5]. Iadecola and colleagues report the expression of ACE2 and transmembrane protease serine (TMPRSS) on the tanycytes and median eminence capillaries [3]. In addition, autopsy studies have elicited the presence of the SARS-CoV-2 genome in the hypothalamus, in addition to highlighting the presence of degenerated and edematous neurons [6]. Furthermore, radiologic imaging has shown evidence of SARS-CoV-2 presence in the brain [7]. The evidence thus suggests that SARS-CoV-2 can breach the brain parenchyma and can potentially cause reversible inflammation-driven hypophysitis or can directly infect the hypothalamus, resulting in hypothalamic-pituitary dysfunction [8].

Our case is that of a young 35-year-old gentleman with no comorbidities. Most patients diagnosed with central DI secondary to COVID-19 were between the ages of 40-70 years [8-12]. However, central DI secondary to COVID-19 has been reported in a two-year-old child by Ghasemi as well [13].

A difference between the onset of central DI from the time of COVID-19 diagnosis has been noted. Yavari A et. al. reported that their patient developed central DI six weeks after being diagnosed with Covid-19 [10]. Misgar et al. illustrated the case of a patient developing central DI eight weeks after contracting Covid-19 [8]. Our patient was a young healthy man who developed central DI on the twelfth day of contracting COVID-19, which is very early in the course of illness and may suggest direct viral infection vs effect of COVID-19-induced immune inflammation.

Patients diagnosed with central DI secondary to COVID-19 often report polyuria. Our patient presented with a high 24-hour urine volume of 4.2 L. In addition, our patient presented with a low urine osmolality, which is expected in central DI secondary to COVID-19. Similar findings of polyuria and low urine osmolality have been reported in the published literature [8-11,13,14].

In central DI, it is estimated that the serum osmolality will be elevated. Yavari et al. and Sheikh et al. reported that patients with central DI secondary to COVID-19 presented with elevated serum osmolalities [10,11]. However, our patient maintained normal serum osmolality.

Another interesting finding in our case is the presence of a low-normal serum sodium level. Such was the case in our patient, who was reported to have a serum sodium level of 136.4 mmol/L. Normal serum sodium levels in central DI secondary to COVID-19 have been reported by Yavari et al. and Sheikh et al. [10,11]. Typically, serum sodium levels are elevated in cases of central DI and such was the case reported by Misgar et al., Rajavec et al., Ghasemi, and Sheikh et al. [8,9,13,14]. Interestingly, hyponatremia in central DI has been reported in the literature by Costa et al. [15]. While dealing with low-normal serum sodium or hyponatremia, the diagnosis of SIADH needs to be considered. The presence of low urine osmolality, increased total urine volume, and positive response to desmopressin suggest the diagnosis of central DI.

Upon further investigation, our patient was found to have an elevation in the urine sodium (252 mmol/24h). Normal levels have been observed in the patient reported by Ghasemi [13]. Sheikh et al. reported a patient with low urine sodium but in another patient reported normal urine sodium levels [11,14]. In our patient, natriuresis can be explained by the pathophysiology of central DI. In central DI, there is a lack of arginine vasopressin (AVP) action on the V2 receptors [16]. V2 receptors are responsible for both water reabsorption and sodium reabsorption [17]. Due to the lack of action at the V2 receptors, both water and sodium are not reabsorbed, resulting in polyuria and natriuresis. As a result, our patient had a normal serum osmolality and a lower normal value of serum sodium than expected. The association between COVID-19 and natriuresis, however, needs to be further investigated.

A dramatic improvement in the patient’s condition was noted upon administration of desmopressin. The published literature reports improvement of the patient’s condition in response to the administration of desmopressin acetate (DDAVP) [8-11,13,14]. Rajavec et al. noted an improvement in the patient’s condition; however, the patient passed away due to complications secondary to COVID-19 [9]. Yavari et al. and Ghasemi reported improvement in the patient’s condition; however, follow-up was not reported [10,13]. Sheikh et al. reported that the patient lost to follow-up [11]. Our patient recovered within one month, and upon follow-up, complete recovery was noted. To our knowledge, ours is the first article to report both a complete follow-up and the timeline under which the patient recovered.

Central DI poses a great risk of fatality when it is at the severe stage, which can take place due to severe dehydration, hypernatremia cardiovascular dysfunction, or failure due to its inability to regulate the hemodynamics (blood pressure) [18]. However, with early identification and treatment with desmopressin, along with regular monitoring and follow-up, complications associated with the condition and the side effects of the drug can be reduced.

The only limitation of our case was the lack of brain imaging. However, the patient refused the same due to financial reasons.

## Conclusions

In conclusion, we report a case of central DI in a 35-year-old male less than two weeks post a COVID-19 infection. The patient did not have any other comorbidities or risk factors for the central DI. His symptoms resolved post-administration of desmopressin, and he made a successful recovery by four weeks. Central DI should be considered one of the top differential diagnoses of any COVID-19 patients presenting with polyuria and polydipsia. We would like to highlight the importance of prompt recognition, diagnosis, and treatment of central DI in a COVID-19 patient. Additionally, we would like to encourage the need for regular follow-up for a successful recovery. Further studies are required to understand if central DI is an active manifestation or an early-onset complication of COVID-19 infection.
